# The regulatory impact of serine/threonine-specific protein phosphorylation among cyanobacteria

**DOI:** 10.3389/fpls.2025.1540914

**Published:** 2025-02-12

**Authors:** Thomas Barske, Martin Hagemann

**Affiliations:** Department Plant Physiology, University of Rostock, Rostock, Germany

**Keywords:** *Synechocystis*, proteomics, kinase, mutant, environment

## Abstract

Cyanobacteria are the only prokaryotes capable of performing oxygenic photosynthesis. To thrive under environmental fluctuations, photosynthesis and metabolic activities needs to be adjusted. Previous studies showed that the acclimation of primary carbon metabolism to fluctuating carbon/nitrogen levels is mainly regulated at post-transcriptional level including diverse posttranslational modifications (PTMs). Protein phosphorylation is regarded as main PTM in the sensing and balancing metabolic changes. In this review we aim to summarize the knowledge on serine/threonine-specific protein phosphorylation among cyanobacteria. Phosphoproteome studies identified several hundred phosphoproteins bearing many more specific phosphorylation sites. On the other hand, only relatively few serine/threonine-specific protein kinases were annotated in cyanobacterial genomes, for example 12 in the model cyanobacterium *Synechocystis* sp. PCC 6803. Systematic mutation of the kinase-encoding genes revealed first insights into their specific functions and substrates. Future research is needed to address how a limited number of protein kinases can specifically modify hundreds of phosphoproteins and to uncover their roles in the regulatory networks of cyanobacterial metabolism.

## 1 Introduction to cyanobacteria

Cyanobacteria are the only prokaryotes performing oxygenic photosynthesis, i.e. they assimilate all organic matter from inorganic nutrients using light energy to produce the necessary energy and reducing power thereby releasing O_2_. In fact, ancient cyanobacteria invented this process at least 2.7 billon years ago, which had a profound impact on the earth geochemistry (Hohmann-Marriott and Blankenship, 2011). Furthermore, chloroplasts found in the kingdom of plantae are derivatives from a single endosymbiotic event approximately 1.5 billion years ago, in which a free-living cyanobacterium was engulfed by an heterotrophic eukaryotic cell (e.g., Weber et al., 2006). Since then, cyanobacteria continued to evolve and through their outstanding capability to adapt to changing environmental conditions inhabited all kinds of diverse habitats in which some light is available (Houmard, 1995). Today, cyanobacteria contributing to almost one quarter of globally fixed CO_2_ and additionally are important assimilators of atmospheric N_2_ (Kasting and Siefert, 2002; Durall and Lindblad, 2015). Lately, through increasing concern about climate change and the exploration for sustainable energy sources raised much interest in photoautotrophic cyanobacteria as chassis to produce biofuels and chemical feedstock (Angermayr et al., 2009; Ducat et al., 2011; Hagemann and Hess, 2018). Thus, bioengineered cyanobacteria were generated to manufacture products such as ethanol (Deng and Coleman, 1999; Kopka et al., 2017), isobutyraldehyde and isobutanol (Atsumi et al., 2009), fatty acids (Liu et al., 2011), sucrose (Ducat et al., 2012; Qiao et al., 2018), and isoprene (Lindberg et al., 2010; Pade et al., 2016). However, production yields remained low making the production of bio-compounds not yet economically feasible (Hagemann and Hess, 2018).

In order to improve the application of cyanobacteria, a deeper understanding of the primary carbon metabolism and its regulation is necessary. As in all other oxygenic phototrophs, cyanobacteria assimilate CO_2_ into organic matter through the Calvin-Benson-Bassham (CBB) cycle, with ribulose 1,5-bisphosphate carboxylase/oxygenase (Rubisco) as the key CO_2_-fixing enzyme. To compensate for the low CO_2_ affinity of Rubisco and its side reaction with O_2_ (Tcherkez et al., 2006), which leads to the production of the toxic byproduct 2-phosphoglycolate that is salvaged in the photorespiratory cycle (Eisenhut et al., 2008a), cyanobacteria evolved the inorganic carbon (Ci, CO_2_ and bicarbonate)-concentrating mechanism (CCM), which increases CO_2_ levels in the proximity of Rubisco. The CCM in *Synechocystis* sp. PCC 6803 comprises three bicarbonate uptake transporters: (i) the constitutively expressed Na^+^-bicarbonate symporter BicA (Price et al., 2004), (ii) the low C_i_ induced ABC-type transporter BCT1 (Omata et al., 1999), and (iii) Na^+^-bicarbonate symporter SbtA (Shibata et al., 2002). Additionally, CO_2_ can be converted into bicarbonate by either the constitutively expressed Ndh1-4 or the low C_i_-induced Ndh1-3 complex (Shibata et al., 2001). Rubisco together with carbonic anhydrase are confined to the carboxysome, in which the accumulated bicarbonate is converted into CO_2_ thereby saturating Rubisco carboxylation and largely inhibiting its oxygenase reaction (Rae et al., 2013; Hagemann et al., 2021).

The fixed carbon is then channeled into different metabolic routes to produce cyanobacterial biomass and reserve polymers (reviewed in Lucius and Hagemann, 2024). Previous studies revealed that cyanobacteria such as *Synechocystis* sp. PCC 6803 cells undergo a global metabolic reprogramming when cultivated under different C/N ratios, e.g., after shifts from high CO_2_ (5%, HC) into ambient air (0.04% CO_2_, LC) (Eisenhut et al., 2008b). Interestingly, the distinct metabolic signature is similar to metabolic changes in the model plant *Arabidopsis thaliana* under low versus high photorespiratory flux (Orf et al., 2016a). However, shifts from HC to LC do not cause significant changes in transcript levels for enzymes involved in primary carbon metabolism (Klähn et al., 2015). Likewise, proteomic studies revealed that carbon metabolism proteins respond more strongly to light changes, but barely to different Ci availability (Jahn et al., 2018; Spät et al., 2021; Barske et al., 2023). Such findings point toward biochemical control rather than transcriptional regulation to enable a quick acclimation of carbon partitioning without comparatively high energetic costs for proteomic responses (Jablonsky et al., 2016). In contrast, the expression of CCM-related genes is under control of transcription factors, namely NdhR (Figge et al., 2001; Wang et al., 2004), CmpR (Omata et al., 2001), and CyAbrB2 (Shalev-Malul et al., 2008; Orf et al., 2016b).

In many studies the unicellular cyanobacterial strain *Synechocystis* sp. PCC 6803 (hereafter *Synechocystis*) have been used as model. This strain was isolated from a freshwater pond in Berkeley and became part of the Pasteur Culture Collection (PCC) in 1968 (Rippka et al., 1979; Zavřel et al., 2017). Although being classified as a freshwater cyanobacterium, *Synechocystis* can be found in a variety of different habitats e.g., coastal areas and even areas of high salinity (Reed and Stewart, 1985; Pattanayak et al., 2015). *Synechocystis* can perform different modes of lifestyle i.e., photoautotrophy, mixotrophy, and light-activated heterotrophy on external glucose (reviewed in Lucius and Hagemann, 2024). As a non-diazotrophic cyanobacterium, it can only grow with combined nitrogen sources, usually with nitrate. *Synechocystis* was the first organism performing oxygenic photosynthesis with a fully sequenced genome (Kaneko et al., 1996; Kaneko and Tabata, 1997). Through its natural competency to take up foreign DNA and incorporate it into its own genome by homologous recombination (Grigorieva and Shestakov, 1982), *Synechocystis* is accessible to genetic manipulation. The early available genome sequence and the genetic systems permitted to generate and characterize a large collection of specific mutants of *Synechocystis*, including collection of different protein kinase-encoding genes. Moreover, new proteomic technologies have been applied to this model strain that uncovered a high number of phosphoproteins that can regulate cyanobacterial metabolism under different growth conditions. Therefore, our review is mostly dealing with results obtained from studying *Synechocystis* and protein-kinase-defective mutants of this strain.

## 2 Regulation of the primary C-metabolism in *Synechocystis*

To accommodate appropriate metabolic fluxes, the primary C-metabolism needs to be able to flexible acclimated itself towards changing environmental conditions, which is mainly performed through biochemical, post-transcriptional regulation (Jablonsky et al., 2016). Because some metabolic routes involve similar enzymes, which can work in opposite directions (reviewed in Lucius and Hagemann, 2024), a multilayered regulatory system is needed to effectively respond to changes in environment and to avoid futile cycles within the metabolic network. However, regulatory mechanisms, particularly on post-transcriptional level of the primary C-metabolism are scarcely understood among cyanobacteria.

During the last years, a few regulatory circuits have been identified that somehow regulate the carbon metabolism in *Synechocystis* and likely other cyanobacteria. The RNA polymerase σ factor SigE can act as a positive regulator of genes involved in carbohydrate catabolism in dark-exposed cells living in a heterotrophic lifestyle and shows a circadian oscillation reaching its peak in light/dark transition (Osanai et al., 2005, 2011). Further mutant studies identified the histidine kinase (Hik) 8 (encoded by *sll0750*), an orthologue to circadian clock protein SasA, to play a role in the control of the C-metabolism (Osanai et al., 2015; Huang et al., 2023). Likely Hik8 interacts with response regulator (Rre) 37 that is encoded by the gene *sll1330* (Osanai et al., 2014). A mutant defective in Rre37 is no longer capable of light-activated heterotrophic growth (Osanai et al., 2014). Furthermore, Hik 37 (*slr0110*) seem to be involved in glucose-mediated catabolism (Gao et al., 2014). The transcription factor RpaA is somehow involved in SigE degradation in the dark and stimulates transcription of enzymes of glycogen and glucose metabolism (Iijima et al., 2015; Köbler et al., 2018). Together with the clock complex KaiAB1C1-SasA, RpaA also affects the switch from autotrophy in the light to the usage of stored carbon in the dark (Scheurer et al., 2021).

Posttranslational modifications i.e. covalent modifications of amino acid side chains, protein-protein interaction and effector-metabolite-binding become of interest as regulatory mechanism in bacterial carbon allocation and have been reported to play an important role in coordinating glycolytic fluxes in animal and plant cells (e.g., Zaffagnini et al., 2013; van Heerden et al., 2015). GlnB (P_II_) is one of the best characterized regulatory proteins in cyanobacteria. By binding 2-oxoglutarate (2OG), the precursor for ammonia assimilation in cyanobacteria, ATP and/or ADP, P_II_ is able to integrate information about the C/N balance and energy state of the cell and adjust C- and N-fluxes accordingly (reviewed in Forchhammer et al., 2022). Recently, P_II_ has been demonstrated to interact with the small protein PirC (Orthwein et al., 2021). PirC is said to be released from P_II_ under N-limitation sensed by increased cellular 2OG levels and interacts with the phosphoglycerate mutates 1 (Pgam1) and thus blocking fluxes into the lower glycolysis and thereby favoring anabolic glycolytic routes and the accumulation of glycogen. Furthermore, P_II_ can be phosphorylated at Ser49 in *Synechocystis* (reviewed in Forchhammer et al., 2022) and the P_II_ phosphorylation state responds to different C/N ratios (e.g., Schwarz et al., 2014). Recently, it was shown that absence of the carbon-metabolism-regulating Hik8 impacts also P_II_ phosphorylation (Huang et al., 2023). The small, disordered protein CP12 known to bind the CBB enzymes GapDH2 and PRK under oxidative conditions is modulating CBB and OPP activity under redox changing conditions (Gurrieri et al., 2021; Lucius et al., 2022). In addition to proteins sensing the redox or metabolic state inside the cyanobacteria, proteome studies revealed an increasing number of post-translational protein modifications (PTMs) on many enzymes involved in the primary C and N metabolism. Among them, protein phosphorylation is regarded to play a central role in the signal recognition and regulation of cellular activities among cyanobacteria as has been shown in many other bacteria (Maček et al., 2019).

## 3 Protein phosphorylation classes among bacteria

Reversible protein phosphorylation and dephosphorylation is one of the most important PTMs that is catalyzed by protein kinases and phosphatases, respectively. Protein kinases are defined as enzymes that transfer a phosphate group onto an amino acid (AA) side chain in a target protein (Cozzone, 1993). Generally, protein kinases using the γ-phosphate of ATP as phosphate group donor but additionally GTP and PEP were shown to serve as phosphate group suppliers (Hunter, 2012). According to the targeted AA, the Nomenclature Committee of International Union of Biochemist classified protein kinases into 5 groups: (i) AA with alcohol groups as acceptors such as serine and threonine forming phosphate esters, (ii) AA with phenolic groups as acceptors namely tyrosine forming phosphate esters, (iii) basic AA such as histidine, arginine and lysine producing phosphoramidates, (iv) AA with acyl groups as acceptors such as aspartate and glutamic acid generating mixed phosphate-carboxylate acid anhydrites, and (v) cysteine residues as acceptor that produce thioesters (Hunter, 2012; Cozzone, 1993).

**Figure 1:**
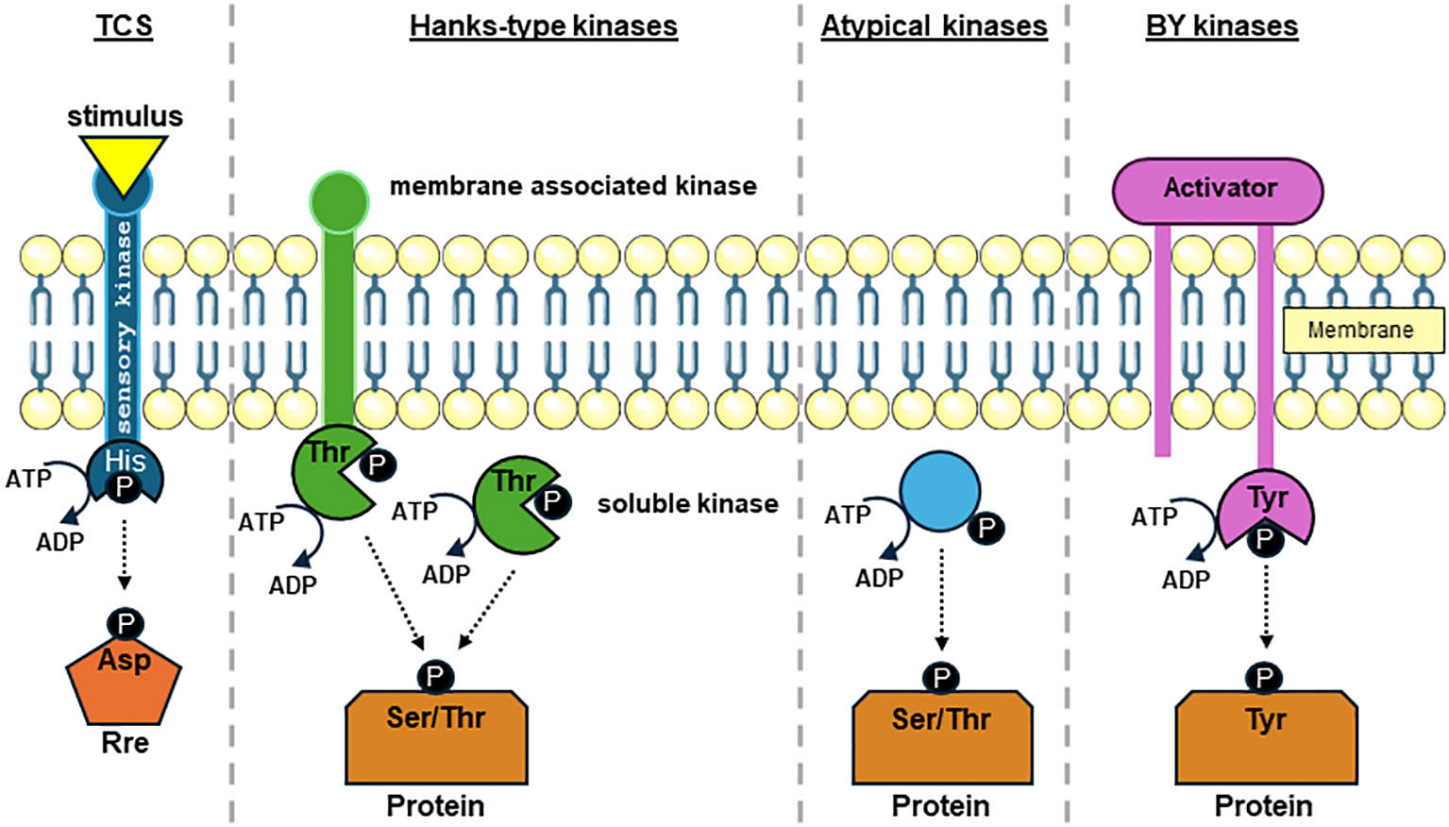
Overview of protein-kinase-mediated phosphate groups transfer to amino acid side chains in bacteria. Phosphate group transfer to side chains of histidine (His), aspartic acid (Asp), serine (Ser), threonine (Thr), and tyrosine (Tyr) residues by prototypic protein kinases using the γ-phosphate of ATP as phosphate group donor is displayed. Two component system (TCS) consist usually of a membrane associated His kinase (Hik) and a response regulator (Rre) that commonly affects the transcription of target genes. Upon sensing an environmental stimulus, autophosphorylation occurs on a His sidechain, which subsequently is transferred to an Asp in the Rre. Bacterial Hanks-kinases can either harbor transmembrane domains or are soluble within the cytosol. Hanks-type kinases can catalyze the phosphate group transfer to both Ser and Thr sidechains. Atypical Ser and Thr protein kinases share no or only marginal similarity to Hanks-kinases. Bacterial tyrosine (BY) kinases require an activator protein to stabilize the kinase complex. When activated, BY kinase autophosphorylate on a Tyr residue and transfers the phosphate group to a Tyr residue on the target protein.

In bacteria, protein phosphorylation by different protein kinase classes (**Figure 1**) is considered as a signal transduction device that links impulses from environmental conditions with the regulation of essential physiological processes (Kobir et al., 2011). Such signals are often transmitted via histidine autophosphorylation and aspartate phosphorylation in two component systems (TCS), which represents the most common type of protein phosphorylation signaling in bacteria and marks the most abundant form of p-events in bacteria (Maček et al., 2019). TCS can be found in all bacterial species and comprise of a signal sensing histidine kinase (Hik). Often membrane associated, upon signal perception Hik use ATP to auto-phosphorylate themselves on a histidine residue which in turn can transfer the phosphate onto an aspartate residue on a response regulator (Rre). The Rre can usually bind on specific promoter sequences thereby translating the sensed signal into a stress-specific response (Stock et al., 2000; Hirakawa et al., 2020). Hence, TCS mediated signal cascades lead to transcriptional changes in most cases (Gross et al., 1989).

Phospho-esters on serine, threonine and tyrosine are the second most common form of protein phosphorylation in bacteria (Maček et al., 2019). Protein phosphorylation upon serine and threonine residues are commonly catalyzed by Hanks-type kinases (**Figure 1**) that share a strong similarity to kinases found in eukaryotes (Hanks et al., 1988; Hanks, 2003; Pereira et al., 2011; Mijakovic et al., 2016; Maček et al., 2019). Hanks-type kinases in bacteria can be membrane bound or can exist as soluble proteins. It should be noted that in addition to Hanks-type kinases other kinases have been described phosphorylating serine and threonine residues termed atypical kinases (Pereira et al., 2011; Mijakovic et al., 2016). Unlike serine and threonine phosphorylation no eukaryotic-like tyrosine kinases were identified in bacteria (Grangeasse et al., 2012). The majority of tyrosine residue phosphorylation is carried out by the protein kinase family bacterial protein-tyrosine kinases (BY-kinases; **Figure 1**). BY-kinases often sense extracellular signals involving an activator protein part (Grangeasse et al., 2012; Mijakovic et al., 2016). Protein arginine phosphorylation was discovered recently in *B. subtilis*, where it was proven to affect factors in the stress response system. This view was only recently expanded by reports of arginine phosphorylation in *S. aureus* (Fuhrmann et al., 2016; Elsholz et al., 2012; Schmidt et al., 2014; Junker et al., 2018). The cysteine thiol-group is not only prone to oxidative modification but can also undergo phosphorylation (Mijakovic et al., 2016). Cysteine phosphorylation has been shown to play a regulatory role in the control of transcription factors in *S. aureus* (Sun et al., 2012).

Finally, protein phosphorylation is often involved in transcriptional regulation using alternative sigma factors in bacteria. In this process anti-sigma factors involved in partner switching systems such as RsbW and SpoIIAB are serine kinase, which are highly conserved among bacterial species and can specifically activate/inactivate alternative sigma factors under specific growth conditions. Such partner switching system belong to the abundant of phosphorylation-events in bacteria (e.g., Bouillet et al., 2018). The kinase activity of RsbW-like proteins such as Slr1861 or PmgA of *Synechocystis* have been verified by *in vitro* phosphorylation assays (Shi et al., 1999; Nakamura et al., 2024). Several proteins involved in sigma factor partner switching systems have been identified as phosphoproteins in *Synechocystis* (**Supplementary Table 1**).

## 4 Protein phosphorylation in cyanobacteria

Cyanobacterial diversity and ability to adapt to changing environmental conditions is highlighted by their complexity of signal perception systems (Zorina, 2013). Thus, rapid signal transmission from a receptor to a receiver is essential for an adequate response to stress. Such dynamics is possible through reversible PTMs for example in form of protein phosphorylation (Maček and Mijakovic, 2011). First direct evidence of protein phosphorylation in cyanobacteria was obtained through [^32^P] orthophosphate *in vivo* and cell-free *in vitro* labeling experiments in *Calothrix* sp. PCC 7601 (Schuster et al., 1984), *Synechococcus* sp. PCC 6301 (Allen et al., 1985), *Anabaena* sp. PCC 7120 (Mann et al., 1991), *Synechocystis* (Bloye et al., 1992; Hagemann et al., 1993), and in *Synechococcus elongatus* PCC 7942 (Forchhammer and Tandeau De Marsac, 1994). These studies showed phosphorylation of many protein bands, which were dynamic under altering growth conditions. However, in most cases the nature of the phosphorylated protein(s) remained enigmatic. The overall physiological importance of protein phosphorylation in cyanobacteria and their role in regulatory processes e.g., nitrogen metabolism, carbon metabolism, cell motility or osmotic stress has been widely acknowledged in later studies as outlined below.

### 4.1 Phosphoproteomics

Even though histidine and aspartate phosphorylation usually account for the majority of p-events in bacteria, their thermodynamic instability renders the *in vivo* detection challenging in phosphoproteomic studies (Maček et al., 2019). The mostly applied extraction of phosphorylated peptides is carried out under acidic conditions (pH <4) and thus favoring the detection of p-events on serine, threonine and tyrosine residues instead of phosphorylated histidine and aspartate residues through their low chemically stability in a lower pH (Maček and Mijakovic, 2011; Mijakovic and Maček, 2012; Maček et al., 2019). Depending for long on quantifying relative proteins levels of perturbed biological systems in 2D-gels, early global phosphoproteome studies relied heavily on this technology to identify p-events (Maček and Mijakovic, 2011). Technical advances in gel-free and mass-spectroscopy (MS)-based approaches resulted in the first published *in vivo* phosphoproteome of *Bacillus subtilis* in an exponential growth phase (Maček et al., 2007a) and of *E. coli* (Maček et al., 2007b), which highly increased the number of detected phosphoproteins. Similar developments occurred among cyanobacteria.

2D-gel-based and gel-free phosphoproteomic experiments revealed an ever-increasing number of phosphoproteins in *Synechocystis.* The first 2D-gel based phosphoproteome study was performed under fluctuating salinity condition (Mikkat et al., 2014). To identify phosphoproteins, the *Synechocystis* proteins were first separated by isoelectric focusing (first dimension) and then in large SDS-gels (second dimension). This separation technique discriminated many proteins and was limited to about 500 proteins visible in separate spots. Protein phosphorylation could initially be identified by specific dyes, which was subsequently verified by MS-based estimation of peptide masses that increased by 80 Da when a phosphate group was added (for more details see Mikkat et al., 2014). This study was able to identify 32 phosphoproteins such as GlnB, Kai proteins and a great number of proteins taking part in the primary C-metabolism (e.g., Pgm, Eno, Gap2). The first gel-free LC-MS-based comparative global phosphoproteome study analyzed the acclimation of *Synechocystis* towards N starvation (Spät et al., 2015). In this approach, the entire proteome is digested with different proteases and then the defined protein fragments are separated by LC techniques. Proteins can subsequently be identified by peptide-mass-fingerprinting via MS. This technique compares the sizes of *in silico* produced proteolytic fragment patterns with the *in vitro* measured peptide sizes, thereby permitting precise protein identification. Again, phosphorylated peptides show an increased mass of 80 Da. To improve the coverage and identification of phosphoproteins, phosporylated peptides are usually enriched by metal-affinity chromatography before the LC separation (for more details see Spät et al., 2015). This study showed an overall increase in p-events under low N conditions, among many proteins were involved in CCM, the primary C-metabolism and the central regulator of C/N partitioning GlnB (P_II_). Similarly, a snapshot phosphoproteome experiment under variating light conditions was performed and discovered that many photosynthesis related proteins undergo changes in p-occupancy in changing light qualities (Chen et al., 2015). Furthermore, this study showed through side specific mutations that the phosphorylation of phycocyanin β-subunit CpcB is of importance, e.g. in state transition. Another phosphoproteome study investigated *Synechocystis* cells acclimated to different carbon conditions with the emphasis on evaluating protein phosphorylation in relation with C-fixation, photosynthesis and photoprotection (Angeleri et al., 2016). Alterations in the proteome and phosphoproteome levels under different growth conditions were also analyzed by Toyoshima et al. (2020). The authors compared photoautotrophic, photoheterotrophic, heterotrophic growth in the presence of light and mixotrophic condition together with growth under N-starvation (24 h, 48 h) and revealed that relatively small alterations in the proteome can be accompanied with vast deviations in overall protein phosphorylation status in the cell and highlights the underlying significance of reversible protein phosphorylation in acclimation processes. We analyzed recently changes in the proteome and phosphoproteome of the *Synechocystis* wild-type and selected kinase mutants under different CO_2_ levels (Spät et al., 2021; Barske et al., 2023). These studies showed that the abundances of enzymes involved in the primary C metabolism remained similar under high and low CO_2_, however, several proteins showed marked changes in their phosphorylation.

Summarizing the phosphoproteome attempts with *Synechocystis* (Mikkat et al., 2014; Spät et al., 2015, 2018, 2021; Chen et al., 2015; Angeleri et al., 2016; Toyoshima et al., 2020; Barske et al., 2023) resulted in a list of at least 481 phosphoproteins (**Supplementary Table 1**), i.e., more than 10% of the entire *Synechocystis* proteome can be phosphorylated under specific growth conditions. Furthermore, many of the identified phosphoproteins displayed more than one phosphorylated site, hence, the total number of phosphorylation events (p-events) on *Synechocystis* is much higher (see also Spät et al., 2023 for a comprehensive overview on proteomic and phosphoproteomic data obtained with *Synechocystis*). Similarly high numbers of phosphoproteins were detected through a global phosphoproteome study with marine cyanobacterium *Synechococcus* sp. PCC 7002 where 410 p-events on 245 proteins could be detected (Yang et al., 2013). Moreover, Liang et al. (2021) analyzed the phosphoproteome of *Nostoc flagelliforme* in response to dehydration. The authors were able to detect 271 phosphoproteins with 1168 phosphorylation sites. Among them, many showed changed phosphorylation under dehydration, especially on proteins known to be involved in signal transduction and response to reactive oxygen species (ROS).

The physiological relevance of the identified p-events remains in most cases elusive. Clear evidence was provided for the importance of protein phosphorylation sites in phosphoglucomutase 1 (Pgm1), which revealed the role of posttranslational modification on serine 47 (S47) during nitrogen starvation and its concomitant role in modulating its activity (Doello et al., 2022). Additionally, it was discovered that regulatory proteins such as P_II_ (Forchhammer and Tandeau De Marsac, 1994) and CP12 (Spät et al., 2018, 2021) are prone to protein phosphorylation and furthermore presenting changes in their phosphorylation status under changing environmental conditions (Forchhammer and Tandeau De Marsac, 1994; Spät et al., 2018). Another early identified phosphoprotein is KaiC, the clock protein involved in the circadian rhythm of *Synechococcus elongatus* PCC 7942 (Nakajima et al., 2005) and many other cyanobacteria including *Synechocystis* (e.g., Köbler et al., 2024). Here, the rate of phosphorylation and dephosphorylation determines the phase of the circadian clock, which is sensed by certain output proteins (Golden et al., 1998). These examples indicate the significance of posttranslational modification in form of protein phosphorylation as a hallmark in metabolic regulation in *Synechocystis* and likely in other cyanobacteria.

### 4.2 Serine/threonine-specific protein kinases in *Synechocystis*

Even though phosphorylation on serine, threonine and tyrosine residues were known to occur in cyanobacteria, studies on kinases creating phospho-monoesters were initially largely neglected (Mann, 1994). This changed after the discovery of a Hanks-type Ser/Thr kinase in *Myxococcus xanthus* (Muñoz-Dorado et al., 1991). Subsequently, similar PCR-based strategies were also employed in cyanobacteria such as *Anabaena* sp. PCC 7120 and resulted in the discovery of the first cyanobacterial Hanks-kinase (Zhang, 1993, 1996) with an increasing number of similar kinases and phosphatases found in other cyanobacteria ever since (Zhang et al., 2005). The available genome sequences revealed that the distribution of Ser/Thr and Tyr kinases and phosphatases is rather uneven among cyanobacteria and can vary from 0, detected in some *Prochlorococcus* strains, to up 56 encoding genes in the nitrogen-fixing strain *N*. *punctiforme* PCC 73102 (Zhang et al., 2005; Zorina, 2013). Interestingly, freshwater cyanobacteria seem to harbor a larger number of Ser/Thr and Tyr kinases and phosphatases compared to marine cyanobacteria, while no clear correlation between genome size and number of Ser/Thr and Tyr kinases and phosphatases could be made (Zhang et al., 2005).

**Figure 2:**
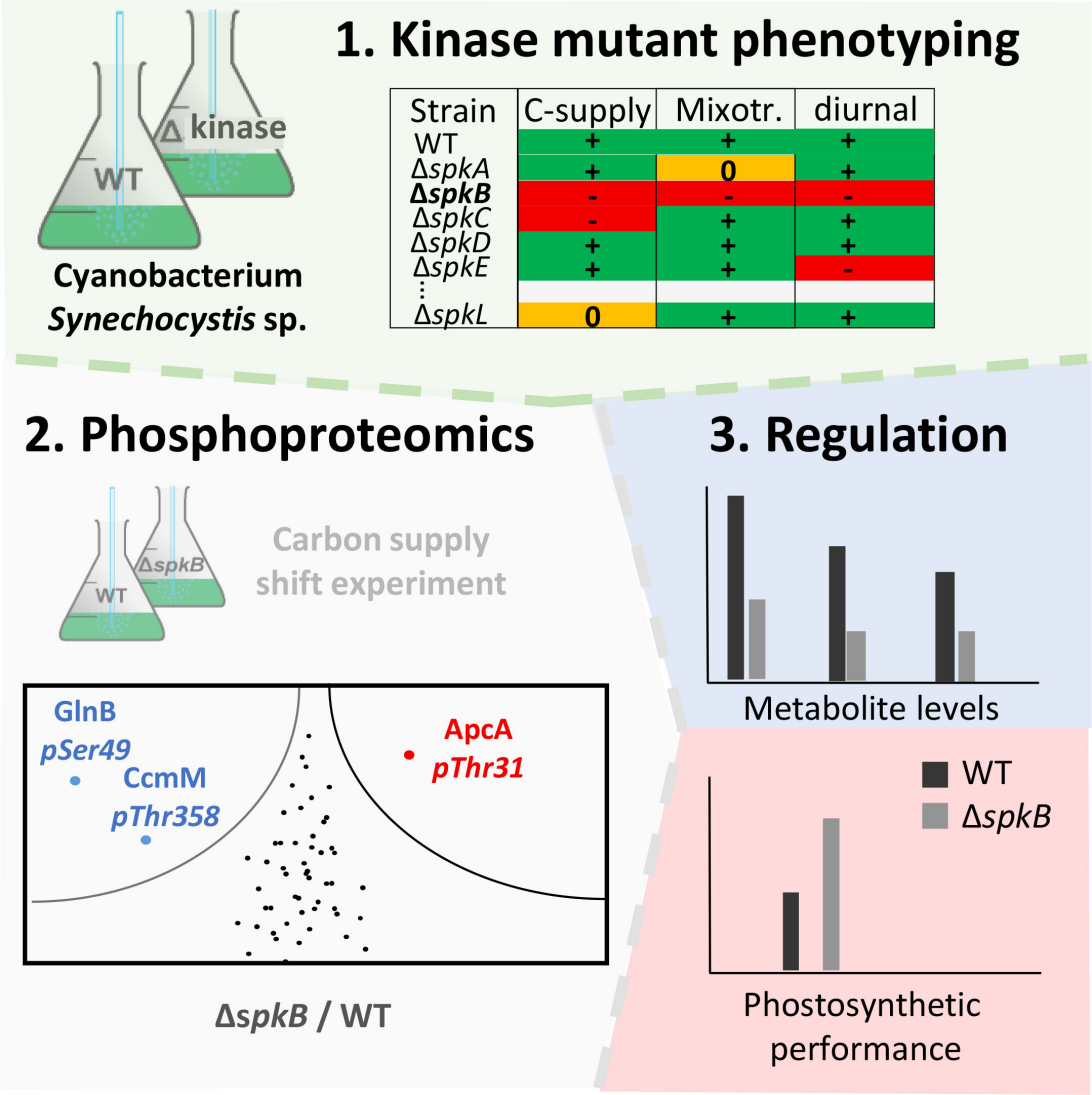
Strategy for the in depth characterization of serine/threonine-specific protein kinases (Spk’s) combining the screening of mutant collections under different growth conditions, applying phosphoproteomics, and subsequent physiological characterizations (e.g., Barske et al., 2023).

Soon after the first cyanobacterial genome sequence of *Synechocystis* was released (Kaneko et al., 1996), it was searched for Ser/Thr and Tyr-specific protein kinases and phosphatases, which bear similarities to Hanks-kinases and Hanks-phosphatases. The search revealed that *Synechocystis* possesses 12 Ser/Thr kinases, one Tyr-kinases and 7 phosphatases (Zhang et al., 1998; Leonard et al., 1998; Shi et al., 1998). The 12 kinases can be divided into “serine/threonine-protein N2-like kinases” - PKN2 and “activity of BC1 complex” kinases - ABC1 (Leonard et al., 1998), respectively (**Table 1**). The PKN2 group comprises the protein kinases SpkA-G and share strong structural similarity to Hanks-kinases (Leonard et al., 1998; Zhang et al., 2007). Protein kinase activity could be verified for SpkA-F using artificial substrates such as histone, MBP and casein as well as autophosphorylation activity with exception of SpkE (Kamei et al., 2001, 2002, 2003). However, Zorina et al. (2014) detected protein kinase activity for SpkE with casein and other substrates as well, hence, all annotated PKN2-type kinases with the exception of SpkG are principally active enzymes. Protein kinases SpkH-L belong to atypical ABC1 protein kinase family (Leonard et al., 1998). Only the catalytic activity of the SpkH was recently confirmed (Zorina et al., 2023). Several groups established collections of protein kinase mutants of *Synechocystis*, which were screened regarding phenotypic alterations and sometimes specific protein substrates during the last years (Kamei et al., 2002; Laurent et al., 2008; Zorina et al., 2011; Mata-Cabana et al., 2012; Barske et al., 2023). The combination of such screening attempts with subsequent phosphoproteomic experiments and physiological measurements permitted the functional characterization of several annotated Spk’s in *Synechocystis* during the last years (**Figure 2**). Basic, kinase-specific features are summarized in **Table 1** and discussed in the next paragraphs.

**Table 1:** Overview on annotated Ser/Thr-specific protein kinases (Spk’s) in *Synechocystis* sp. PCC 6803 and some related features.

Kinase	Type	Gene	Active	Mutant*	Phenotype(s)
SpkA	Pkn2	*sll1574/75*	yes^1^	yes^1,6^	Non-motile^1^
SpkB	Pkn2	*slr1697*	yes^2^	yes^2,6,8^	Non-motile^2^ No mixotrophic growth^5,6^ Sensitive against methyl-viologen^5^ More resistant against H_2_O_2_^6^ Slower growth under low CO_2_^6^
SpkC	Pkn2	*slr0599*	yes^3^	yes^3,6,8,12^	Increased tolerance towards methylamine^10^ Slower growth under low CO_2_^6^
SpkD	Pkn2	*sll0776*	yes^3^	no^3^/yes^6,8^	Pleiotropic effects, no growth at ambient CO_2_ and at high CO_2_ in the presence of ammonia^11^
SpkE	Pkn2	*slr1443*	yes^7^	yes^3,6,8^	Involved in expression of cold-shock proteins^7^
SpkF	Pkn2	*slr1255*	yes^3^	yes^3,6,8^	Almost wild-type like^6^
SpkG	Pkn2	*slr0152*	n.i.	yes^4,6,8^	Sensitive to 855 mM NaCl^4^
SpkH	ABC1	*sll0005*	yes^9^	no^6^/yes^8^	Only essential kinase according to ^6^
SpkI	ABC1	*sll1770*	n.i.	yes^6,8^	Pleiotropic effects, sensitive to many stresses^6^
SpkJ	ABC1	*slr0889*	n.i.	yes^6,8^	Almost wild-type like^6^
SpkK	ABC1	*slr1919*	n.i.	yes^6,8^	Slower growth under low CO_2_^6^
SpkL	ABC1	*sll0095*	n.i.	yes^6,8^	Almost wild-type like^6^

*, yes; i.e., completely segregated; *, no; i.e., only partly segregated; n.i., not investigated; 1, Kamei et al., 2001; 2, Kamei et al., 2003; 3, Kamei et al., 2002; 4, Liang et al., 2011; 5, Mata-Cabana et al., 2012; 6, Barske et al., 2023; 7, Zorina et al., 2014; 8, Zorina et al., 2011; 9, Zorina et al., 2023; 10, Galkin et al., 2003; 11, Laurent et al., 2008; 12, Spät et al., 2021.

#### 4.2.1 SpkA

The *spkA* encoding sequence is found on two separate genes in the primarily sequenced glucose-tolerant *Synechocystis* wild type (**Table 1**), whereas it forms a continuous gene in the original glucose-sensitive wild type from the Pasteur Culture Collection. Inactivation of the wild-type *spkA* gene provided evidence of its role in cell motility, because the null mutant Δ*spkA* showed no colony movement under lateral illumination (Kamei et al., 2001). Though retaining pili, the authors proposed that SpkA might not be essential for pili biogenesis but can somehow influence gliding motility towards a light source. This view was further expanded by a gene transcriptome analysis with Δ*spkA* in which expression changes for pilin encoding operons were detected. Some of them were subsequently verified in Northern-blot experiments showing reduced level of *pilA9* and enhanced quantities of *pilA5* together with the observation of missing thick pili (Panichkin et al., 2006). Our recent survey of phenotypic variations among *spk* mutants revealed slower growth of Δ*spkA* under mixotrophic and salt stress conditions compared to wild type (Barske et al., 2023).

#### 4.2.2 SpkB

Initial reports for the SpkB-deficient mutant Δ*spkB* described a similar phenotype as Δ*spkA* with a strongly reduced gliding motility (Kamei et al., 2003). Additionally, it was demonstrated that the absence of SpkB resulted in a redox-sensitive phenotype, because in contrast to wild type the mutant was unable to tolerate elevated concentrations of menadion, methylviologen or high light doses, i.e. treatments that induce high intracellular levels of ROS. Furthermore, ^32^P-labelling of protein extracts revealed a decrease of one of the most prominent phosphorylated protein bands, which mainly contained GlyS (glycyl-tRNA synthetase subunit beta) and thus might represent one specific substrate of SpkB (Mata-Cabana et al., 2012).

Our recent experiments with the mutant Δ*spkB* verified the non-motile phenotype, which is correlated by the differential accumulation of many proteins associated with the cell surface including pili subunits (Barske et al., 2023). The most striking differences of Δ*spkB* compared to the *Synechocystis* wild type were related to carbon metabolism, because mutant cells grew significantly slower under low CO_2_, while high CO_2_ conditions complemented the phenotype. Furthermore, the mutant Δ*spkB* was sensitive to glucose additions in the light and under diurnal light rhythms, which is correlated with changes in glycogen accumulation (Barske et al., 2023). The detailed analysis of the proteome revealed only few alterations in protein abundances, none of them is directly correlated with the changed ability to grow at ambient CO_2_ or in the presence of external glucose. However, the phosphoproteome revealed that two proteins were not phosphorylated anymore in the mutant. To this end, phosphorylation of the proteins Sll1545 and Slr0483 was only detected in wild-type samples. Slr1545 is the glutathione S-transferase, Gst1, which plays an important role in the redox regulation of proteins among cyanobacteria (Kammerscheit et al., 2019). Its expression change could be related to the observed differences in ROS-tolerance of Δ*spkB*. The Slr0483 protein is a membrane protein of unknown function that bears a CAAD domain (cyanobacterial aminoacyl-tRNA synthetase appended domain, PMID: 18775859). In addition to these proteins without any detected phosphorylation in Δ*spkB*, a few p-events were identified with significantly diminished phosphorylation (Barske et al., 2023). Among them, the (auto)phosphorylation of SpkF at T24 was significantly diminished under ambient CO_2_ in the mutant Δ*spkB* accompanied with generally reduced SpkF levels in this strain. Furthermore, the phosphorylation of the P_II_ (GlnB) protein at S49 was strongly reduced in Δ*spkB* compared to the WT when grown at high CO_2_ and especially when shifted for 3 h to ambient CO_2_. The P_II_ protein is the master regulator of many aspects in the C/N homeostasis in cyanobacteria and other organisms as well (reviewed in Forchhammer et al., 2022). But no protein involved in the CCM or the glucose/glycogen metabolism showed any significant changes in abundance and phosphorylation levels, which make it difficult to explain the observed phenotypic alteration of mutant Δ*spkB* under different carbon conditions (Barske et al., 2023). Finally, it should be noted that our study detected only non-phosphorylated GlyS in extracts of the wild type and the mutant Δ*spkB*, while a previous study identified GlyS as substrate for SpkB (Mata-Cabana et al., 2012).

#### 4.2.3 SpkC

Several studies were conducted on the characterization of SpkC, which is encoded by *slr0599*. Initially it was reported that mutant Δ*spkC* shows an increased tolerance toward the toxic compounds methylamine and methionine-sulfoximine in low light, while several other kinase mutants behaved like wild type in this study (Galkin et al., 2003). A microarray experiment with wild-type cells exposed to the inhibitors DCMU and DBMIB in low light characterized an induction of *spkC* transcription (Hihara et al., 2003). Zorina et al. (2011) analyzed the mutant Δ*spkC* and several others in a 2D-gel-based phosphoproteome study under standard and heat shock conditions. They found rather minor changes in the overall proteome but several changes in the phosphorylation of protein spots, among them some heat-shock proteins including GroES. Subsequently, they used recombinant GroES as substrate in *in vitro* phosphorylation assays with crude extracts from several *spk* mutants. In contrast to wild type and other *spk* mutants, extracts from mutant Δ*spkC* and Δ*spkF as well as* Δ*spkK* showed no GroES phosphorylation, whereas crude protein extracts from the corresponding complementation strains were able to phosphorylate GroES. According to these results they proposed a cascade of SpkC/F/K in phosphorylating the heat-shock protein GroES (Zorina et al., 2011).

More recently, we re-investigated the phosphoproteome of the mutant Δ*spkC* under high and low CO_2_, because it showed a diminished growth after shifts from high to ambient CO_2_ levels (Spät et al., 2021). Overall, more than 2500 proteins were quantified in our study, equivalent to approximately 70% of the *Synechocystis* theoretical proteome. Proteins with changing abundances under different CO_2_ levels are often involved in the CCM or the nitrogen metabolism, whereas enzymes related to primary carbon metabolism showed almost no changes in their abundances. Interestingly, among the few proteins with changed abundances the bicarbonate transporter SbtA and some other low-CO_2_-induced proteins were less strongly accumulated in mutant Δ*spkC* than in wild type, which is consistent with the slower growth of the mutant at ambient conditions (Spät et al., 2021). Furthermore, 105 phospho-proteins harboring over 200 site-specific phosphorylation events were identified. Subunits of the bicarbonate transporter BCT1 and the redox switch protein CP12 were among phosphoproteins with reduced phosphorylation levels at lower CO_2_, whereas the serine/threonine protein kinase SpkC revealed increased phosphorylation levels, which supports its possible regulatory involvement in the acclimation towards changing CO_2_ conditions. To identify potential target proteins of SpkC-mediated phosphorylation, we searched for phosphoproteins that were reproducibly identified in wild type but were never detected in the mutant Δ*spkC*. According to this attempt, at least four potential phosphorylation targets of SpkC were identified. Among them, the phosphorylation in the subunit CmpB of the ATP-dependent bicarbonate transporter BCT1 was always absent in Δ*spkC*, whereas it was detectable in every replicate from wild type. This phosphorylation change might be directly connected to lowered growth of mutant Δ*spkC* under lowered CO_2_. In addition, phosphorylation of the DnaJ-like protein Sll1384, of Slr1619, and of the response-regulator-like protein Slr6040 on plasmid pSYSX occurred exclusively in the wild type. Collectively, our data make it likely that SpkC is somehow involved in the sensing/regulation of the acclimation of cyanobacteria towards limiting CO_2_ conditions. In this regard it is interesting to note that SpkC was identified as integral protein in the *Synechocystis* plasma membrane (Liberton et al., 2016).

#### 4.2.4 SpkD

In an early study it was shown that SpkD might be essential for *Synechocystis* to grow under standard laboratory conditions, because only partially deletion of the *spkD-*encoding *sll0776* gene was achieved (Kamei et al., 2002). However, in later studies it was possible to receive completely segregated Δ*spkD* mutants (Laurent et al., 2008; Barske et al., 2023), which make the essential character of the kinase questionable. These different results can be related to slight differences in the wild-type strains of *Synechocystis* used in the corresponding studies, however, it cannot be excluded that still unknown suppressor mutations allowed the segregation in the later studies. Laurent et al. (2008) observed a clear upregulation of *spkD* expression under low CO_2_ compared to high CO_2_ and concomitantly Δ*spkD* was unable to grow under ambient air. The authors suggested that SpkD is involved in adjustments of TCA cycle metabolites, because the supplementation of alternative organic carbon sources such as glucose, phosphoglyceraldehyde and pyruvate could not rescue the mutant phenotype, whereas the external addition of TCA metabolites e.g., acetyl CoA, succinate, and 2OG were able to revert it (Laurent et al., 2008). However, in our recent survey of many *spk* mutants, we did not observe very clear alterations in mutant Δ*spkD* compared to wild type, including its wild-type-like growth under low and elevated CO_2_ levels (Barske et al., 2023). Recent transcriptome analysis found *spkD* upregulated in the *lexA* mutant (Kizawa et al., 2016). Moreover, phosphoproteome studies under different light (Chen et al., 2015) conditions and our results under changing Ci availability (Spät et al., 2021) identified SpkD as a phosphoprotein. Finally, SpkD together with SpkG were hypothesized to influence the expression of fatty acid desaturases, i.e., they might play a role in the regulation of PUFA contents (Chen et al., 2021).

#### 4.2.5 SpkE

The PKN2-type kinase SpkE is encoded by the gene *slr1443* in *Synechocystis* and was verified to be an active protein kinase (**Table 1**). An early study reported that SpkE seems be required for post-translational modification of pili proteins after biogenesis (Kim et al., 2004). Another study provided some evidence that SpkE might be involved in cold shock response (Zorina et al., 2014). Cells of the mutant Δ*spkE* showed the most prominent change in the expression of cold-shock proteins when shifted from 32°C to 22°C for 30 min. In addition to the protein synthesis pattern, the 2D-gel-based approach showed also significant changes in the protein phosphorylation patterns. Some of these changes were also observed in the mutant Δ*hik33*, a sensory histidine-kinase proven to be involved in cold shock and other stress responses. These finding led to the hypothesis that SpkE might be an additional component in cold stress responses in *Synechocystis* (Zorina et al., 2014). In our study, the mutant Δ*spkE* showed diminished growth under diurnal conditions, but the mechanism underlying this phenotype was not further investigated (Barske et al., 2023).

#### 4.2.6 SpkF

Induction of *spkF* expression was observed in a transcriptome study analyzing ethanol resistance in *Synechocystis* (Wang et al., 2012). Furthermore, SpkF has been shown to be prone to modulation by phosphorylation upon N-starvation (Spät et al., 2015) with a reported transiently increase in phosphorylation of SpkF when *Synechocystis* resuscitates after chlorosis (Spät et al., 2018) and under changes in Ci availability (Spät et al., 2021). Our recent phosphoproteome study identified a diminished (auto)phosphorylation of SpkF at T24 under ambient CO_2_ in the mutant Δ*spkB* accompanied with generally reduced SpkF levels in this strain, however, the ability of mutant Δ*spkF* to grow at elevated or ambient CO_2_ conditions was not changed (Barske et al., 2023). A global proteome study identified SpkF as integral proteins in the plasma membrane (Liberton et al., 2016).

#### 4.2.7 SpkG

In contrast to most other PKN2-type kinases, the enzymatic activity of SpkG has not been verified. The *spkG* gene is transcribed as last gene in the photosystem II assembly protein operon (*slr0144-slr0152*), in which several phosphoproteins have been identified (Angeleri et al., 2016). An upcoming study provided indirect evidence that SpkG might represent an active protein kinase, because in the mutant Δ*spkG* the phosphorylation of some proteins, especially ferredoxin 5 (Fd5) did not occur anymore, hence, it could be concluded that SpkG might be specifically involved in Fd5 phosphorylation (Angeleri et al., 2018). Moreover, *spkG* expression was strongly induced when *Synechocystis* was exposed to elevated salt concentrations and, accordingly, growth of Δ*spkG* was impaired in the presence of 855 mM NaCl (Liang et al., 2011), whereas the mutant Δ*spkG* grew like wild type on plates supplemented with 500 mM NaCl (Barske et al., 2023). The possible involvement of SpkG in the sensing of salt stress was supported by transcriptome studies, which showed that some high-salt-induced genes are less strongly expressed in the mutant Δ*spkG* (Liang et al., 2011). More recently, SpkG together with SpkD were hypothesized to influence the expression of fatty acid desaturases, i.e. they might play a role in the regulation of PUFA contents (Chen et al., 2021). Finally, transcriptomics showed that in H_2_O_2_-treated wild-type cells *spkG* was found to be downregulated (Li et al., 2004).

#### 4.2.8 SpkH

In our survey of different *spk* mutants, we were not able to completely segregate the mutation of *sll0005* encoding SpkH despite several attempts (Barske et al., 2023), whereas Zorina et al. (2011) reported a complete knock out of this gene. As mentioned above, the different results can be related to slight differences in the wild type strains of *Synechocystis* used in the corresponding studies, however, it cannot be excluded that still unknown suppressor mutations allowed the segregation in the earlier study. Recently, its enzymatic activity was verified, because recombinant SpkH protein was able to phosphorylate some typical protein kinase substrate proteins (Zorina et al., 2023). The *spkH* expression was reported to be light induced and significantly downregulated after addition of salt (Allakhverdiev et al., 2002). Correspondingly, *spkH* was also found to be highly expressed in *Synechocystis* under osmotic stress in from of supplemented sorbitol (Paithoonrangsarid et al., 2004).

#### 4.2.9 SpkI

Among the ABC1-type kinases, SpkI received most attentions in *Synechocystis* (Irmler and Forchhammer, 2001; Huang et al., 2002; Wang et al., 2022). This high interest is related to the localization of the SpkI encoding gene *sll1770*, which is situated upstream of the gene for the P_II_ phosphatase *pphA.* Hence, it was initially suspected that SpkI might be involved in P_II_ phosphorylation, because the P_II_-specific kinase is still not identified. But it was proven that SpkI is non-essential for the phosphorylation of the P_II_ protein (Irmler and Forchhammer, 2001). A global gene expression study displayed a *spkI* induction under UV-B and intense light intensities (Huang et al., 2002). More recent reports highlighted a wild-type-like growth accompanied with a higher NPQ capacity in Δ*spkI* under standard growth condition (Wang et al., 2022). However, the mutant showed reduced growth and decreased net photosynthesis in a high salt environment. Moreover, reduced levels of major photosynthetic protein were detected while fluorescence measurements revealed modification in photosystem I and Cytb6f-complex together with impaired Q_A_ and state transition. Hence the authors suggested a central role of SpkI in maintaining photosynthesis during salt acclimation (Wang et al., 2022). A reduced capability of mutant Δ*spkI* to grow under diverse stress conditions such as changes in the CO_2_ level, diurnal rhythms, and salt stress was also found in our recent survey of kinase mutants (Barske et al., 2023). This pleiotropic phenotype might be related to the important role of SpkI in regulation of photosynthesis.

#### 4.2.10 SpkJ

To our knowledge, no conclusive data are available for SpkJ.

#### 4.2.11 SpkK

SpkK could be located to the thylakoid membrane (Baers et al., 2019) and is proposed to act in a cascade together with SpkC and SpkF in the phosphorylation of the heat shock protein GroES (Zorina et al., 2011). We reported a slower growth of mutant Δ*spkK* when transferred from high to ambient CO_2_ conditions (Barske et al., 2023).

#### 4.2.12 SpkL

The function of SpkL has not been analyzed in great detail. The *spkL* gene showed lower expression under iron starvation (Hernández-Prieto et al., 2012).

## 5 Conclusions and outlook

Much progress has been made in the field of cyanobacterial phosphoproteins and protein kinases. Especially the technical advances in phosphoproteomics resulted in an increasing number of identified phosphoproteins in *Synechocystis* (**Supplementary Table 1**) and other cyanobacteria as well. However, it must be mentioned that compared to the MS-based protein identification and quantification, which permits the quantitative detection of approximately 75% of the theoretical *Synechocystis* proteome (Spät et al., 2023), the detection and quantification of phosphoproteins is much less reliable. In many cases, phosphorylated peptides need to be enriched through specific affinity media, which make the method less reproducible and difficult to absolute quantification. In our recent studies, we applied identical growth, protein extraction and proteome methods, which resulted in a consistent list of quantified proteins (almost 95% reproduction) in the *Synechocystis* wild type and selected mutants in the two independent studies (Spät et al., 2021; Barske et al., 2023). In contrast, the list of identified phosphoproteins and their CO_2_-dependent changes in phosphorylation levels was much less reproducible, only approximately 50% of the phosphorylated proteins were found in the two studies. Furthermore, the detection limit of present-day MS methods improved many times, which makes it possible to detect also rare p-events, i.e. phosphorylated amino acid residues with less than 1% occupancy. But, the physiological meaning of such rare protein phosphorylation is highly questionable.

Nevertheless, the high number of detected phosphoproteins, many of them showed changes in the phosphorylation under different growth conditions, is consistent with the assumption that protein phosphorylation represents the dominant PTM involved in regulation of metabolism and stress acclimation. However, only in a relatively few cases, we have solid biochemical or physiological evidence that the changes in protein phosphorylation indeed affected enzyme activities or photosynthetic performance (examples are mentioned and discussed above). In the majority of cases, we can only speculate which or even if any function is related to the observed protein phosphorylation. Obviously, many more studies are needed in which specific protein variants with and without existing phosphorylation sites are studied *in vivo* and *in vitro* in detail. Such studies should include the identification of the responsible protein kinases and also phosphatases. In most cases the responsible kinases for a specific p-event are unknown. Even many proposed functions for the studied protein kinases are only evidence based. *In vitro* assays proofing the direct interaction with their claimed targets remains to be conducted in future experiments, for example with recombinant SpkB to verify its specific involvement in the phosphorylation of GlnB as proposed by Barske et al. (2023).

One of the most obvious open question is related to the large discrepancy between the high number of phosphoproteins and the even higher number of p-events (**Table 1**; Spät et al., 2023) and the much smaller number of Ser/Thr protein kinases in *Synechocystis* (**Table 1**). Similar large deviations between hundreds of p-events and small numbers of annotated protein kinases have been reported for other bacteria as well, which initiated attempts to use computational predictions to analyze the kinase/substrate interactions (reviewed in Grunfeld et al., 2024). Generally, this situation makes it difficult to assume very specific kinase/protein substrate interactions in bacteria such as *Synechocystis*. Hence, it is generally assumed that bacterial Spks have a rather relaxed substrate specificity and the same kinase can act in different regulatory mechanisms together with other proteins (Kobir et al., 2011; Grunfeld et al., 2024). Moreover, in addition to kinase mediated p-events, kinase independent p-events are possible, as it has recently been shown for the phosphorylation of PGM1 and PGM2 (Doello et al., 2022; Neumann et al., 2022). Those events could vastly increase the total number of p-events. Furthermore, the complete picture of Ser/Thr protein kinases in *Synechocystis* should be re-evaluated. Many bacterial Ser/Thr protein kinases resemble Hanks-type kinases which is also true for *Synechocystis* (Zhang et al., 1998; Leonard et al., 1998; Shi et al., 1998). Nevertheless, there is a chance of evolutionary unrelated protein kinases such as the isocitrate dehydrogenase kinase/phosphatase found in *E. coli* (Zheng and Jia, 2010) or the non-enzymatic acetyl phosphate dependent phosphorylation which was reported in the bacteria *Streptococcus pneumoniae* (Kaiser et al., 2020) and *B. subtilis* (Cairns et al., 2015). Hence, many more efforts are necessary to uncover the complex role of protein phosphorylation in stress acclimation and metabolic control in cyanobacteria.
