# Supplementary material for: The regulatory impact of serine/threonine-specific protein phosphorylation among cyanobacteria
Source: Front Plant Sci. 2025 Feb 12;16:1540914. doi: 10.3389/fpls.2025.1540914 (PMC11863333; doi:10.3389/fpls.2025.1540914)
Supplement: Supplementary file 1 [file Table1.docx]

**Supplementary Table 1**: List of identified phosphoproteins based on published global phosphoproteome studies with *Synechocystis* sp. PCC 6803 cultivated under a wide range of conditions (Mikkat et al., 2014; Spät et al., 2015, 2018, 2021; Chen et al., 2015; Angeleri et al., 2016; Toyoshima et al., 2020; Barske et al., 2023).

| **Cyanobase ID** | **Name** | **Protein Function** | **Cyanobase ID** | **Name** | **Protein Function** |
| --- | --- | --- | --- | --- | --- |
| *sll0017* | HemL | glutamate-1-semialdehyde aminomutase | *slr0171* | Ycf37 | photosystem I assembly related protein Ycf37 |
| *sll0018* | FbaA | fructose-bisphosphate aldolase, class II | *slr0185* | PyrE | orotate phosphoribosyltransferase |
| *sll0019* | Dxr | 1-deoxy-d-xylulose 5-phosphate reductoisomerase | *slr0186* | LeuA | 2-isopropylmalate synthase |
| *sll0039* | PixH | positive phototaxis protein, two-component response regulator CheY subfamily | *slr0192* | Slr0192 | hypothetical protein |
| *sll0040* | PixI | positive phototaxis protein, homologous to chemotaxis protein CheW | *slr0193* | Rbp31 | RNA-binding protein |
| *sll0041* | PixJ | phytochrome-like photoreceptor protein for positive phototaxis;homologous to methyl-accepting chemotaxis protein | *slr0213* | GuaA | GMP synthetase |
| *sll0042* | PixJ2; Tar | methyl-accepting chemotaxis protein for positive phototaxis | *slr0228* | FtsH2 | cell division protein FtsH |
| *sll0043* | PixL | positive phototaxis protein, homologous to chemotaxis protein CheA, two-component hybrid histidine kinase | *slr0242* | Bcp | bacterioferritin comigratory protein homolog |
| *sll0057* | GrpE | heat shock protein GrpE | *slr0261* | NdhH | NADH dehydrogenase subunit 7 |
| *sll0068* | Sll0068 | unknown protein | *slr0288* | GlnN | glutamate--ammonia ligase |
| *sll0094* | Hik37 | two-component sensor histidine kinase | *slr0301* | PpsA | phosphoenolpyruvate synthase |
| *sll0103* | Sll0103 | hypothetical protein | *slr0322* | Hik43 | two-component hybrid sensor and regulator |
| *sll0108* | Amt1 | ammonium/methylammonium permease | *slr0335* | ApcE | phycobilisome core-membrane linker polypeptide LCM |
| *sll0135* | MtnP | putative 5'-methylthioadenosine phosphorylase | *slr0342* | PetB | cytochrome b6 |
| *sll0142* | Sll0142 | probable cation efflux system protein | *slr0363* | Slr0363 | hypothetical protein |
| *sll0144* | PyrH | uridine monophosphate kinase | *slr0374* | Slr0374 | hypothetical protein |
| *sll0163* | Sll0163 | WD-repeat protein | *slr0377* | Slr0377 | unknown protein |
| *sll0172* | Sll0172 | periplasmic protein, function unknown | *slr0384* | SqdX | sulfoquinovosyldiacylglycerol biosynthesis protein SqdX |
| *sll0173* | Vgb | virginiamycin B hydrolase, periplasmic protein | *slr0393* | #N/A | #N/A |
| *sll0182* | Sll0182 | ABC transporter ATP-binding protein | *slr0394* | Pgk | phosphoglycerate kinase |
| *sll0185* | Sll0185 | hypothetical protein | *slr0397* | Slr0397 | hypothetical protein |
| *sll0199* | PetE | plastocyanin | *slr0404* | Slr0404 | hypothetical protein |
| *sll0205* | Sll0205 | hypothetical protein | *slr0426* | FolE | GTP cyclohydrolase I |
| *sll0207* | RfbA | glucose-1-phosphate thymidylyltransferase | *slr0434* | Efp | elongation factor P |
| *sll0217* | Flv4 | flavoprotein Flv4 | *slr0447* | UrtA | periplasmic protein, ABC-type urea transport system substrate-binding protein |
| *sll0225* | Sll0225 | unknown protein | *slr0452* | IlvD | dihydroxyacid dehydratase |
| *sll0236* | Sll0236 | unknown protein | *slr0453* | Slr0453 | hypothetical protein |
| *sll0245* | Sll0245 | probable GTP binding protein | *slr0455* | Slr0455 | hypothetical protein |
| *sll0258* | PsbV | cytochrome c550 | *slr0459* | Slr0459 | hypothetical protein |
| *sll0260* | Sll0260 | hypothetical protein | *slr0476* | Slr0476 | unknown protein |
| *sll0271* | NusB | N utilization substance protein B homolog | *slr0483* | Slr0483 | hypothetical protein |
| *sll0301* | Sll0301 | hypothetical protein | *slr0503* | Ycf66 | hypothetical protein YCF66 |
| *sll0320* | Rnd | probable ribonuclease D | *slr0536* | HemE | uroporphyrinogen decarboxylase |
| *sll0329* | Gnd | 6-phosphogluconate dehydrogenase | *slr0543* | TrpB | tryptophan synthase beta subunit |
| *sll0359* | CyAbrB | CyAbrB, putative regulatory protein | *slr0546* | TrpC | indole-3-glycerol phosphate synthase |
| *sll0370* | CarB | carbamoyl-phosphate synthase, pyrimidine-specific, large chain | *slr0549* | Asd | aspartate beta-semialdehyde dehydrogenase |
| *sll0377* | Mfd | transcription-repair coupling factor | *slr0551* | Slr0551 | hypothetical protein |
| *sll0379* | LpxA | acyl-[acyl-carrier-protein]-UDP-N-acetylglucosamine o-acyltransferase | *slr0552* | Slr0552 | hypothetical protein |
| *sll0408* | Sll0408 | peptidyl-prolyl cis-trans isomerase | *slr0557* | ValS | valyl-tRNA synthetase |
| *sll0416* | GroL2 | 60 kDa chaperonin 2, GroEL2, molecular chaperone | *slr0559* | NatB | periplasmic binding protein of ABC transporter for natural amino acids |
| *sll0430* | HtpG | HtpG, heat shock protein 90, molecular chaperone | *slr0585* | ArgG | argininosuccinate synthetase |
| *sll0446* | Sll0446 | unknown protein | *slr0599* | SpkC | serine/threonine kinase SpkC |
| *sll0469* | PrsA | ribose-phosphate pyrophosphokinase | *slr0601* | Slr0601 | unknown protein |
| *sll0497* | Sll0497 | hypothetical protein | *slr0609* | Slr0609 | hypothetical protein |
| *sll0505* | Sll0505 | hypothetical protein | *slr0617* | Slr0617 | unknown protein |
| *sll0508* | Sll0508 | unknown protein | *slr0623* | TrxA | thioredoxin |
| *sll0513* | Sll0513 | hypothetical protein | *slr0637* | Slr0637 | hypothetical protein |
| *sll0517* | RbpA | putative RNA binding protein | *slr0645* | Slr0645 | hypothetical protein |
| *sll0518* | Sll0518 | unknown protein | *slr0649* | MetS | methionyl-tRNA synthetase |
| *sll0529* | Sll0529 | hypothetical protein | *slr0654* | Slr0654 | unknown protein |
| *sll0541* | DesC (des9) | acyl-lipid desaturase (delta 9) | *slr0661* | ProC | pyrroline-5-carboxylate reductase |
| *sll0542* | AcsA | acetyl-coenzyme A synthetase | *slr0670* | Slr0670 | hypothetical protein |
| *sll0550* | Flv3 | flavoprotein Flv3 | *slr0676* | CysC | adenylylsulfate kinase |
| *sll0563* | Sll0563 | unknown protein | *slr0686* | Slr0686 | hypothetical protein |
| *sll0567* | Fur | ferric uptake regulation protein | *slr0700* | Slr0700 | probable amino acid permease |
| *sll0569* | RecA | RecA gene product | *slr0708* | Slr0708 | periplasmic protein, function unknown |
| *sll0576* | HrEpiB | putative sugar-nucleotide epimerase/dehydratease | *slr0721* | Me | malic enzyme |
| *sll0585* | Sll0585 | hypothetical protein | *slr0729* | Slr0729 | hypothetical protein |
| *sll0602* | Sll0602 | hypothetical protein | *slr0730* | Slr0730 | hypothetical protein |
| *sll0617* | Vipp1 | plasma membrane protein essential for thylakoid formation | *slr0737* | PsaD | photosystem I subunit II |
| *sll0634* | BtpA | photosystem I biogenesis protein BtpA | *slr0744* | InfB | translation initiation factor IF-2 |
| *sll0635* | ThiE | probable thiamine-phosphate pyrophosphorylase | *slr0752* | Eno | enolase |
| *sll0654* | Sll0654 | alkaline phosphatase | *slr0755* | Slr0755 | hypothetical protein |
| *sll0660* | PdxA | pyridoxal phosphate biosynthetic protein PdxA | *slr0758* | KaiC | circadian clock protein KaiC homolog |
| *sll0680* | PstS | phosphate-binding periplasmic protein precursor (PBP) | *slr0772* | ChlB | light-independent protochlorophyllide reductase subunit ChlB |
| *sll0726* | Pgm | phosphoglucomutase | *slr0820* | #N/A | #N/A |
| *sll0744* | Sll0744 | hypothetical protein | *slr0833* | DnaB | replicative DNA helicase [Contains: Ssp dnaB intein] |
| *sll0756* | Sll0756 | unknown protein | *slr0875* | MscL | large-conductance mechanosensitive channel |
| *sll0764* | UrtD | urea transport system ATP-binding protein | *slr0882* | Ycf84 | hypothetical protein YCF84 |
| *sll0776* | SpkD | serine/threonine kinase SpkD | *slr0884* | Gap1 | glyceraldehyde 3-phosphate dehydrogenase 1 (NAD+) |
| *sll0778* | #N/A | #N/A | *slr0891* | AmiA | N-acetylmuramoyl-L-alanine amidase |
| *sll0788* | #N/A | #N/A | *slr0906* | PsbB | photosystem II core light harvesting protein |
| *sll0813* | CtaC | cytochrome c oxidase subunit II | *slr0925* | Ssb | single-stranded DNA-binding protein |
| *sll0819* | PsaF | photosystem I reaction center subunit III precursor (PSI-F), plastocyanin (cyt c553) docking protein | *slr0929* | Slr0929 | chromosome partitioning protein, ParA family |
| *sll0822* | CalB | hypothetical protein | *slr0942* | Slr0942 | alcohol dehydrogenase [NADP+] |
| *sll0851* | PsbC | photosystem II CP43 protein | *slr0943* | Fda | fructose-bisphosphate aldolase, class I |
| *sll0854* | Sll0854 | hypothetical protein | *slr0947* | RpaB | response regulator for energy transfer from phycobilisomes to photosystems |
| *sll0863* | #N/A | #N/A | *slr0952* | Fbp | fructose-1,6-bisphosphatase |
| *sll0865* | #N/A | #N/A | *slr0963* | Sir | ferredoxin-sulfite reductase |
| *sll0877* | Sll0877 | hypothetical protein | *slr0982* | RfbB | probable polysaccharide ABC transporter ATP binding subunit |
| *sll0897* | #N/A | DnaJ protein, heat shock protein 40, molecular chaperone | *slr1044* | McpA | methyl-accepting chemotaxis protein, required for the biogenesis of thick pilli |
| *sll0899* | GlmU | UDP-N-acetylglucosamine pyrophosphorylase | *slr1046* | TatA | putative TatA protein |
| *sll0901* | PurE | phosphoribosylaminoimidazole carboxylase | *slr1048* | Slr1048 | hypothetical protein |
| *sll0915* | PqqE | periplasmic protease | *slr1055* | ChlH | magnesium protoporphyrin IX chelatase subunit H |
| *sll0920* | Ppc | phosphoenolpyruvate carboxylase | *slr1063* | Slr1063 | probable glycosyltransferase |
| *sll0922* | #N/A | #N/A | *slr1067* | GalE | UDP-glucose 4-epimerase |
| *sll0923* | EpsB | unknown protein | *slr1096* | LpdA | dihydrolipoamide dehydrogenase |
| *sll0927* | MetX | S-adenosylmethionine synthetase | *slr1102* | Slr1102 | hypothetical protein |
| *sll0928* | ApcD | allophycocyanin-B | *slr1103* | Slr1103 | hypothetical protein |
| *sll0934* | CcmA | carboxysome formation protein CcmA | *slr1104* | Slr1104 | hypothetical protein |
| *sll0947* | LrtA | light repressed protein A homolog | *slr1123* | Gmk | guanylate kinase |
| *sll0981* | Sll0981 | unknown protein | *slr1129* | Rne | ribonuclease E |
| *sll0982* | Sll0982 | unknown protein | *slr1130* | RnhB | ribonuclease HII |
| *sll0996* | MiaB | hypothetical protein | *slr1133* | ArgH | L-argininosuccinate lyase |
| *sll0998* | RbcR | LysR family transcriptional regulator | *slr1137* | CtaD | cytochrome c oxidase subunit I |
| *sll1020* | Sll1020 | probable glycosyltransferase | *slr1160* | Slr1160 | periplasmic protein, function unknown |
| *sll1028* | CcmK2 | carbon dioxide concentrating mechanism protein CcmK | *slr1161* | Slr1161 | hypothetical protein |
| *sll1028;sll1029* | #N/A | #N/A | *slr1163* | Slr1163 | unknown protein |
| *sll1029* | CcmK1 | carbon dioxide concentrating mechanism protein CcmK | *slr1166* | Slr1166 | UDP-glucose:tetrahydrobiopterin glucosyltransferase |
| *sll1031* | CcmM | carbon dioxide concentrating mechanism protein CcmM, putative carboxysome structural protein | *slr1176* | GlgC | glucose-1-phosphate adenylyltransferase |
| *sll1033* | Sll1033 | probable protein phosphatase | *slr1184* | #N/A | #N/A |
| *sll1039* | Sll1039 | hypothetical protein | *slr1225* | SpkF | serine/threonine kinase SpkF |
| *sll1043* | Pnp | polyribonucleotide nucleotidyltransferase | *slr1237* | #N/A | #N/A |
| *sll1059* | Adk2 | adenylate kinase | *slr1261* | Slr1261 | hypothetical protein |
| *sll1070* | TktA | transketolase | *slr1270* | Slr1270 | periplasmic protein, function unknown |
| *sll1074* | LeuS | leucyl-tRNA synthetase | *slr1272* | Slr1272 | probable porin; major outer membrane protein |
| *sll1076* | ZiaA | cation-transporting ATPase, Zinc exporter | *slr1274* | PilM | probable fimbrial assembly protein PilM, required for motility |
| *sll1089* | Sll1089 | periplasmic protein, function unknown | *slr1280* | NdhK1 | NADH dehydrogenase subunit NdhK |
| *sll1096* | RpsL | 30S ribosomal protein S12 | *slr1281* | NdhJ | NADH dehydrogenase subunit I |
| *sll1097* | RpsG | 30S ribosomal protein S7 | *slr1282* | #N/A | #N/A |
| *sll1099* | Tuf | elongation factor Tu | *slr1289* | Icd | isocitrate dehydrogenase (NADP+) |
| *sll1101* | RpsJ | 30S ribosomal protein S10 | *slr1295* | FutA1 | iron transport system substrate-binding protein |
| *sll1106* | Sll1106 | hypothetical protein | *slr1300* | Slr1300 | similar to 2-octaprenyl-6-methoxyphenol hydroxylase |
| *sll1130* | Sll1130 | unknown protein | *slr1311* | #N/A | #N/A |
| *sll1184* | #N/A | heme oxygenase | *sll1867* | #N/A | #N/A |
| *sll1185* | HemF | coproporphyrinogen III oxidase, aerobic (oxygen-dependent) | *slr1325* | SpoT | GTP pyrophosphokinase |
| *sll1188* | Sll1188 | hypothetical protein | *slr1329* | AtpB | ATP synthase beta subunit |
| *sll1194* | PsbU | photosystem II 12 kDa extrinsic protein | *slr1334* | ManB | phosphoglucomutase/phosphomannomutase |
| *sll1213* | Sll1213 | GDP-fucose synthetase | *slr1338* | Slr1338 | hypothetical protein |
| *sll1214* | #N/A | hypothetical protein YCF59 | *slr1349* | Pgi | glucose-6-phosphate isomerase |
| *sll1217* | Sll1217 | unknown protein | *slr1356* | Rps1A | 30S ribosomal protein S1 |
| *sll1242* | Sll1242 | hypothetical protein | *slr1390* | FtsH1 | cell division protein FtsH |
| *sll1244* | RplI | 50S ribosomal protein L9 | *slr1435* | PmbA | PmbA protein homolog |
| *sll1247* | Sll1247 | hypothetical protein | *slr1459* | ApcF | phycobilisome core component |
| *sll1260* | RpsB | 30S ribosomal protein S2 | *slr1463* | FusA | elongation factor EF-G |
| *sll1275* | Pyk2 | pyruvate kinase 2 | *slr1476* | PyrB | aspartate carbamoyltransferase |
| *sll1283* | SpoIID | similar to stage II sporulation protein D | *slr1505* | Slr1505 | unknown protein |
| *sll1292* | Sll1292 | two-component response regulator CheY subfamily | *slr1512* | SbtA | sodium-dependent bicarbonate transporter |
| *sll1294* | PilJ | methyl-accepting chemotaxis protein | *slr1513* | SbtB | periplasmic protein, function unknown |
| *sll1306* | Sll1306 | periplasmic protein, function unknown | *slr1516* | SodB | superoxide dismutase |
| *sll1307* | Sll1307 | periplasmic protein, function unknown | *slr1517* | LeuB | 3-isopropylmalate dehydrogenase |
| *sll1314* | DctP | putative C4-dicarboxylase binding protein, periplasmic protein | *slr1529* | Slr1529 | nitrogen assimilation regulatory protein |
| *sll1316* | PetC2 | cytochrome b6-f complex iron-sulfur subunit (Rieske iron sulfur protein) | *slr1531* | Ffh | signal recognition particle protein |
| *sll1324* | AtpF | ATP synthase B chain (subunit I) of CF(0) | *slr1536* | RecQ | ATP-dependent DNA helicase RecQ |
| *sll1325* | AtpD | ATP synthase delta chain of CF(1);ATP synthase delta chain of CF(1) | *slr1588* | Slr1588 | two-component transcription regulator |
| *sll1326* | AtpA | ATP synthase alpha chain | *slr1603* | Slr1603 | hypothetical protein |
| *sll1330* | Sll1330 | two-component system response regulator OmpR subfamily | *slr1619* | Slr1619 | hypothetical protein |
| *sll1334* | Sll1334 | two-component sensor histidine kinase | *slr1622* | Ppa | soluble inorganic pyrophosphatase |
| *sll1338* | Sll1338 | unknown protein | *slr1624* | Slr1624 | hypothetical protein |
| *sll1341* | Bfr | bacterioferritin | *slr1634* | Slr1634 | hypothetical protein |
| *sll1342* | Gap2 | NAD(P)-dependent glyceraldehyde-3-phosphate dehydrogenase | *slr1641* | ClpB1 | ClpB protein |
| *sll1350* | Sll1350 | hypothetical protein | *slr1643* | PetH | ferredoxin-NADP oxidoreductase (FNR) |
| *sll1360* | DnaX | DNA polymerase III subunit gamma/tau [Contains: Ssp dnaX intein] | *slr1655* | PsaL | photosystem I subunit XI |
| *sll1362* | IleS | isoleucyl-tRNA synthetase | *slr1657* | Slr1657 | hypothetical protein |
| *sll1363* | IlvC | ketol-acid reductoisomerase | *slr1659* | Slr1659 | hypothetical protein |
| *sll1367* | Sll1367 | hypothetical protein | *slr1665* | DapF | diaminopimelate epimerase |
| *sll1384* | Sll1384 | similar to DnaJ protein | *slr1686* | Slr1686 | hypothetical protein |
| *sll1396* | Sll1396 | unknown protein | *slr1693* | Slr1693 | two-component response regulator PatA subfamily |
| *sll1398* | Psb28 | photosystem II reaction center 13 kDa protein | *slr1697* | SpkB | serine/threonine kinase SpkB |
| *sll1426* | Sll1426 | unknown protein | *slr1704* | Slr1704 | hypothetical protein |
| *sll1434* | MrcA | penicillin-binding protein | *slr1710* | MrcB | penicillin-binding protein |
| *sll1450* | NrtA | nitrate/nitrite transport system substrate-binding protein | *slr1718* | ComB | hypothetical protein |
| *sll1451* | NrtB | nitrate/nitrite transport system permease protein | *slr1729* | KdpB | potassium-transporting P-type ATPase B chain |
| *sll1464* | Sll1464 | hypothetical protein | *slr1735* | BgtA | ATP-binding subunit of the ABC-type Bgt permease for basic amino acids and glutamine |
| *sll1469* | Sll1469 | hypothetical protein | *slr1744* | AmiA | N-acetylmuramoyl-L-alanine amidase, periplasmic protein |
| *sll1479* | Pgl | 6-phosphogluconolactonase | *slr1751* | Prc | periplasmic carboxyl-terminal protease |
| *sll1499* | GlsF | ferredoxin-dependent glutamate synthase | *slr1756* | GlnA | glutamate--ammonia ligase |
| *sll1503* | #N/A | #N/A | *slr1763* | Slr1763 | probable methyltransferase |
| *sll1525* | Prk | phosphoribulokinase | *slr1783* | Ycf29 | two-component response regulator, NarL subfamily |
| *sll1526* | Sll1526 | hypothetical protein | *slr1788* | Slr1788 | unknown protein |
| *sll1536* | MoeB | molybdopterin biosynthesis MoeB protein | *slr1789* | Slr1789 | unknown protein |
| *sll1541* | Sll1541 | hypothetical protein | *slr1793* | TalB | transaldolase |
| *sll1542* | Sll1542 | hypothetical protein | *slr1796* | Slr1796 | hypothetical protein |
| *sll1545* | Gst1 | glutathione S-transferase | *slr1816* | Slr1816 | hypothetical protein |
| *sll1547* | Sll1547 | hypothetical protein | *slr1834* | PsaA | P700 apoprotein subunit Ia |
| *sll1553* | PheT | phenylalanyl-tRNA synthetase | *slr1835* | PsaB | P700 apoprotein subunit Ib |
| *sll1559* | Sll1559 | soluble hydrogenase 42 kD subunit | *slr1841* | Slr1841 | probable porin;major outer membrane protein |
| *sll1573* | Sll1573 | hypothetical protein | *slr1843* | Zwf | glucose 6-phosphate dehydrogenase |
| *sll1577* | CpcB | phycocyanin beta subunit | *slr1844* | UvrA | excinuclease ABC subunit A |
| *sll1578* | CpcA | phycocyanin alpha subunit | *slr1856* | Slr1856 | anti-sigma factor antagonist Slr1856; phosphoprotein substrate of *icfG* gene cluster |
| *sll1579* | CpcC2 | phycobilisome rod linker polypeptide LR30 | *slr1859* | Slr1859 | anti-sigma f factor antagonist Slr1859; phosphoprotein substrate of *icfG* gene cluster |
| *sll1580* | CpcC1 | phycobilisome rod linker polypeptide | *slr1870* | Slr1870 | hypothetical protein |
| *sll1595* | KaiC2 | circadian clock protein, KaiC homolog | *slr1874* | Ddl | D-alanine--D-alanine ligase |
| *sll1600* | #N/A | #N/A | *slr1894* | Slr1894 | probable DNA-binding stress protein |
| *sll1621* | AhpC | AhpC/TSA family protein | *slr1920* | Slr1920 | unknown protein |
| *sll1626* | LexA | LexA repressor | *slr1924* | Slr1924 | D-alanyl-D-alanine carboxypeptidase, periplasmic protein |
| *sll1656* | Sll1656 | hypothetical protein | *slr1939* | HtpX | unknown protein |
| *sll1665* | Sll1665 | unknown protein | *slr1942* | KaiC3 | circadian clock protein KaiC homolog |
| *sll1672* | Hik12 | two-component hybrid sensor and regulator | *slr1945* | PGAM | 2,3-bisphosphoglycerate-independent phosphoglycerate mutase |
| *sll1692* | Sll1692 | hypothetical protein | *slr1950* | CtaA | copper-transporting P-type ATPase CtaA |
| *sll1693* | Sll1693 | hypothetical protein | *slr1963* | OCP | water-soluble carotenoid protein OCP |
| *sll1694* | HofG | pilin polypeptide PilA1 | *slr1983* | Slr1983 | two-component hybrid sensor and regulator |
| *sll1712* | Hup | DNA binding protein HU | *slr1984* | Rps1b | nucleic acid-binding protein, 30S ribosomal protein S1 homolog |
| *sll1734* | CupA | protein involved in low CO_2_-inducible, high affinity CO_2_ uptake | *slr1986* | ApcB | allophycocyanin beta subunit |
| *sll1742* | NusG | transcription antitermination protein NusG | *slr1991* | CyaA | adenylate cyclase |
| *sll1746* | RplL | 50S ribosomal protein L12 | *slr1992* | Gpx2 | glutathione peroxidase-like NADPH peroxidase |
| *sll1747* | AroC | chorismate synthase | *slr1994* | FabG2 | PHA-specific acetoacetyl-CoA reductase |
| *sll1757* | Sll1757 | hypothetical protein | *slr2002* | CphA | cyanophycin synthetase |
| *sll1758* | GlmM | MrsA protein homolog | *slr2003* | Slr2003 | hypothetical protein |
| *sll1761* | Sll1761 | unknown protein | *slr2024* | Slr2024 | two-component response regulator CheY subfamily |
| *sll1787* | RpoB | RNA polymerase beta subunit | *slr2032* | Ycf23 | hypothetical protein YCF23 |
| *sll1789* | RpoC2 | RNA polymerase beta prime subunit | *slr2051* | CpcG | phycobilisome rod-core linker polypeptide LRC |
| *sll1801* | RplW | 50S ribosomal protein L23 | *slr2058* | TopA | DNA topoisomerase I |
| *sll1802* | RplB | 50S ribosomal protein L2 | *slr2067* | ApcA | allophycocyanin alpha subunit |
| *sll1803* | RplV | 50S ribosomal protein L22 | *slr2070* | Slr2070 | hypothetical protein |
| *sll1804* | RpsC | 30S ribosomal protein S3 | *slr2072* | IlvA | L-threonine deaminase |
| *sll1808* | RplE | 50S ribosomal protein L5 | *slr2073* | SepF | hypothetical protein YCF50 |
| *sll1811* | RplR | 50S ribosomal protein L18 | *slr2075* | GroES | 10kD chaperonin |
| *sll1812* | RpsE | 30S ribosomal protein S5 | *slr2076* | GroL1 | 60kD chaperonin |
| *sll1815* | Adk1 | adenylate kinase | *slr2077* | #N/A | #N/A |
| *sll1816* | RpsM | 30S ribosomal protein S13 | *slr2094* | Fbpl | fructose-1,6-/sedoheptulose-1,7-bisphosphatase |
| *sll1821* | RplM | 50S ribosomal protein L13 | *slr2098* | Hik21 | two-component hybrid sensor and regulator |
| *sll1823* | PurA | adenylosuccinate synthetase | *slr2102* | FtsY | cell division protein FtsY |
| *sll1830* | Sll1830 | unknown protein | *slr2115* | #N/A | #N/A |
| *sll1833* | FtsI | penicillin-binding protein | *slr2118* | #N/A | #N/A |
| *sll1841* | OdhB | pyruvate dehydrogenase dihydrolipoamide acetyltransferase component (E2) | *slr5016* | #N/A | #N/A |
| *sll1852* | Ndk | nucleoside diphosphate kinase | *slr5018* | Slr5018 | hypothetical protein |
| *sll1862* | Sll1862 | unknown protein | *slr5051* | Slr5051 | unknown protein |
| *sll1863* | Sll1863 | unknown protein | *slr6001* | #N/A | #N/A |
| *sll1865* | PrfB | peptide chain release factor 2 | *slr6012* | #N/A | #N/A |
| *sll1867* | #N/A | #N/A | *slr6028* | #N/A | #N/A |
| *sll1873* | Sll1873 | unknown protein | *slr6031/slr6090* | #N/A | #N/A |
| *sll1879* | Crr23 | two-component response regulator | *slr6039* | #N/A | #N/A |
| *sll1893* | HisF | cyclase | *slr6040* | #N/A | #N/A |
| *sll1908* | SerA | D-3-phosphoglycerate dehydrogenase | *slr6050* | Slr6050 | hypothetical protein |
| *sll1911* | Sll1911 | hypothetical protein | *slr6106* | Slr6106 | hypothetical protein |
| *sll1921* | Sll1921 | hypothetical protein | *slr6110* | Slr6110 | putative signalling protein |
| *sll1931* | GlyA | serine hydroxymethyltransferase | *slr7010* | #N/A | #N/A |
| *sll1932* | DnaK3 | DnaK protein | *slr7060* | Slr7060 | hypothetical protein |
| *sll1945* | Dxs | 1-deoxyxylulose-5-phosphate synthase | *slr8016* | ParB | plasmid partitioning protein, ParB |
| *sll1967* | Sll1967 | probable RNA methyltransferase | *ssl0483* | Ssl0483 | hypothetical protein |
| *sll1973* | PlsY | hypothetical protein | *ssl0563* | PsaC | photosystem I subunit VII |
| *sll2012* | RpoD; SigD | group2 RNA polymerase sigma factor SigD | *ssl0601* | RpsU | 30S ribosomal protein S21 |
| *sll5042* | Sll5042 | probable sulfotransferase | *ssl0707* | GlnB | nitrogen regulatory protein PII |
| *sll5059* | Crr43 | two-component response regulator | *ssl1046* | Ssl1046 | hypothetical protein |
| *sll5066* | Sll5066 | probable plasmid partitioning protein, ParA family | *ssl1498* | Ssl1498 | hypothetical protein |
| *sll5079* | Sll5079 | probable short chain dehydrogenase | *ssl1552* | Ssl1552 | unknown protein |
| *sll7063* | Sll7063 | unknown protein | *ssl1633* | HliC | high light-inducible polypeptide HliC, CAB/ELIP/HLIP superfamily |
| *sll8031* | #N/A | #N/A | *ssl1707* | Ssl1707 | hypothetical protein |
| *slr0009* | CbbL | ribulose bisphosphate carboxylase large subunit | *ssl1784* | RpsO | 30S ribosomal protein S15 |
| *slr0012* | CbbS | ribulose bisphosphate carboxylase small subunit | *ssl2084* | AcpP | acyl carrier protein |
| *slr0032* | IlvE | probable branched-chain amino acid aminotransferase | *ssl2420* | Ssl2420 | unknown protein |
| *slr0038* | Slr0038 | hypothetical protein | *ssl2982* | RpoZ | DNA-directed RNA polymerase omega subunit |
| *slr0040* | CmpA | bicarbonate transport system substrate-binding protein | *ssl3093* | CpcD | phycobilisome small rod linker polypeptide LR10 |
| *slr0041* | CmpB | bicarbonate transport system permease protein | *ssl3177* | RepA | hypothetical protein |
| *slr0067* | Mrp | MRP protein homolog | *ssl3335* | SecE | preprotein translocase SecE subunit |
| *slr0072* | RsmG | glucose inhibited division protein B | *ssl3364* | CP12 | CP12 polypeptide |
| *slr0073* | Hik36 | two-component sensor histidine kinase | *ssl3445* | RpmE | 50S ribosomal protein L31 |
| *slr0075* | Ycf16 | ABC transporter, ATP-binding protein | *ssl5113* | Ssl5113 | unknown protein |
| *slr0076* | Slr0076 | hypothetical protein | *ssr0680* | #N/A | #N/A |
| *slr0096* | #N/A | #N/A | *ssr1176* | #N/A | #N/A |
| *slr0121* | Slr0121 | hypothetical protein | *ssr1528* | Ssr1528 | hypothetical protein |
| *slr0145* | Slr0145 | unknown protein | *ssr1600* | Ssr1600 | similar to anti-sigma f factor antagonist |
| *slr0148* | Fed5 | ferredoxin-like protein | *ssr1789* | HliD | CAB/ELIP/HLIP-related protein HliD |
| *slr0149* | Slr0149 | hypothetical protein | *ssr2708* | #N/A | #N/A |
| *slr0151* | Slr0151 | unknown protein | *ssr2723* | Ssr2723 | hypothetical protein |
| *slr0152* | SpkG | serine/threonine kinase SpkG | *ssr2831* | PsaE | photosystem I subunit IV |
| *slr0161* | PilT | twitching motility protein PilT | *ssr3189* | Ssr3189 | hypothetical protein |
| *slr0164* | ClpR | ATP-dependent Clp protease proteolytic subunit | *ssr3383* | ApcC | phycobilisome small core linker polypeptide LC |
| *slr0165* | ClpP3 | ATP-dependent Clp protease proteolytic subunit | *ssr3451* | PsbE | cytochrome b559 alpha subunit |
| *slr0168* | Slr0168 | unknown protein |  |  |  |
